# Diagnostic Dilemma of Widespread Vesiculobullous Lesions: A Case Report

**DOI:** 10.31729/jnma.7118

**Published:** 2022-01-31

**Authors:** Nisha Khadka, Subij Shakya, Dikshya Khatiwada, Pravash Budhathoki, Tulsi Ram Bhattarai

**Affiliations:** 1 Kathmandu Medical College and Teaching Hospital, Sinamangal, Kathmandu, Nepal; 2 Department of Internal Medicine, Bronx Lebanon Hospital, USA; 3 Department of Internal Medicine, Kathmandu Medical College and Teaching Hospital, Sinamangal, Kathmandu, Nepal

**Keywords:** *case report*, *cutaneous lupus erythematosus*, *rheumatology*, *stevens-johnson syndrome*

## Abstract

Stevens-Johnson syndrome and toxic epidermal necrolysis represent a spectrum of severe mucocutaneous reactions, while Acute Cutaneous Lupus Erythematosus is a variant of Systemic Lupus Erythematosus. Both are rare conditions, with significant morbidity and mortality; often indistinguishable clinically and pose a diagnostic dilemma for the clinician. We hereby present a unique case of a 17 years old female who presented with widespread vesiculobullous lesions with peeling, desquamation, and crusting of the skin surface, non-scarring alopecia, oral and nasal ulcers, as well as two episodes of generalized tonic-clonic seizures. The patient had a history of intake of itraconazole tablets for a week, 25 days before the disease manifestation.

## INTRODUCTION

Stevens-Johnson syndrome (SJS) and toxic epidermal necrolysis (TEN) are a group of severe cutaneous life-threatening reactions, predominantly drug-induced, with mortality rates as high as 30%.^1^ The annual incidence of SJS and TEN varies from 1-6 per million people and 0.4-1.2 per million people respectively.^2^ TEN-like acute cutaneous lupus erythematosus (TEN-like ACLE) is a rare manifestation of Systemic Lupus Erythematosus (SLE).^3^ TEN-like ACLE has an annual incidence of 4 cases per 100,000 people.^4^ TEN-like ACLE mimics TEN clinically, causing diagnostic dilemma as with our case of a 17 years old female with widespread vesiculobullous lesions.^5^

## CASE REPORT

**Figure 1:**
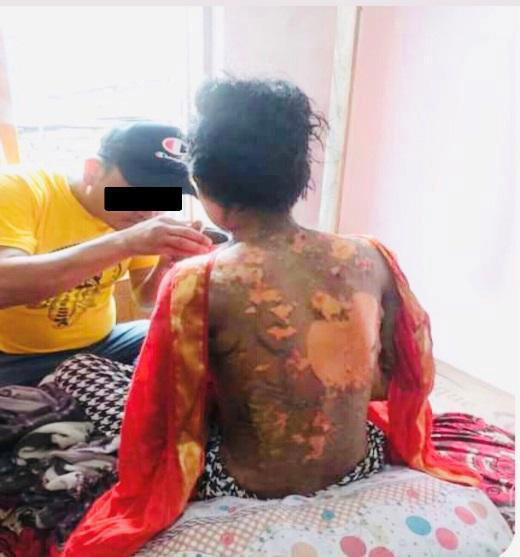
Blisters leading to the desquamation of skin involving the trunk.

A 17 years patient developed multiple well-circumscribed, flat, erythematic, itchy, and painful rashes on the face and upper extremities, mostly on the cheeks and fingertips. She took itraconazole, vitamin-B complex, and zinc tablets from a nearby local pharmacy for a week. Then, she developed irregularly shaped erythematous rashes with blistering over her face, upper limbs, and thighs, with swelling of lips. She stopped taking the medication. The lesions didn't progress as much and eventually regressed. However, in 20 days, she developed widespread erythematous blisters over cheeks, chin, trunk, and thighs. The patient was managed in the hospital outpatient department and was given Inj. methylprednisolone 80mg intramuscularly stat; tab. amoxicillin-clavulanic acid 625mg 8 hourly, tab. ebastine 20mg at night time along with Dermamoist lotion. However, the lesion continued to progress into widespread vesiculobullous lesions with desquamation, peeling of skin into sheets, and crusting of skin surface throughout the affected part, and the general condition of the patient deteriorated with malaise, confusion, and generalized weakness (Figure 1).

Two days later, she presented to the emergency room, where along with the aforementioned skin manifestations and positive Nikolsky sign; she had unstable vital (Blood pressure 80/40mm of Hg, pulse 67bpm, and Spo_2_ 96%), two episodes of generalized tonic-clonic seizure, yellowish sclera, multiple hemorrhagic crusts of upper and lower lips. She had abnormal laboratory parameters like liver function test (Alanine Transaminase: 78U/L; Aspartate Transaminase: 1012U/L; Alkaline phosphatase: 97U/L; serum albumin: 21g/L); Lactate Dehydrogenase: 831U/L; Prothrombin Time (PT) 15 second, PT/international normalized ratio: 1.12sec and pancytopenia. She was admitted to the Intensive Care Unit. Three days later she was diagnosed with SLE based on Systemic Lupus Collaborating Clinics (SLICC) criteria. Under SLICC criteria, the patient fulfills the following parameters:

1. Widespread vesiculobullous lesions with peeling, desquamation, and crusting of skin surface consistent with Acute Cutaneous Lupus
2. Non-scarring alopecia
3. Oral and nasal ulcer
4. Neurological manifestations like two episodes of generalized tonic-clonic seizure and Computed Tomography revealed cerebral cortical atrophy with bilateral prominent cortical sulci, Sylvian fissure, cerebrospinal Fluid spaces the nd Basal Cistern
5. Hemolytic anemia (Hemoglobin- 5.8gm/dl)
6. Thrombocytopenia (70000 platelets per microliter)
7. Leucopenia (3000 leukocytes per microliter)
8. Renal manifestation of proteinuria (urine albumin 3+)and
9. Immunological criteria: Positive Antinuclear Antibody (ANA) by Enzyme-linked immunoassay (ELISA) with the value of 52IU/ml and ANA profile reveals strong positive values for Ribosomal Protein (RIB).

The patient was managed judiciously with steroids, namely intravenous hydrocortisone 200mg 8 hourly, later changed to tab. Prednisolone, gradually tapered and stopped; intravenous antibiotics; meticulous wound care with Clobetasol Propionate, Fusidic acid, and Betamethasone valerate ointment, Aloe vera gel with vitamin A and E; and other symptomatic treatment. The patient gradually became stable.

**Figure 2:**
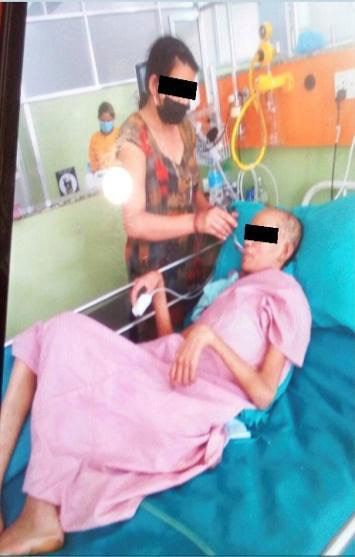
Visible muscle wasting during treatment.

Notwithstanding, the patient developed wasting of muscles with generalized weakness; fluctuating orientation; crying spells with adjustment disorder; grade 2 bedsore was; and was unable to perform twoway communication (Figure 2).

For which, necessary consultations from psychiatry, neurology, plastic surgery, physiotherapy, and Nutrition experts were done and managed as per their expert opinion. The patient was discharged on the 58^th^ day of admission with adequate counseling and medication for residual issues.

## DISCUSSION

A diagnostic dilemma arises in a case like this. The TEN-like variant of SLE is believed to occur in patients with acute or subacute Cutaneous Lupus Erythematosus. It typically develops features of TEN with unusual subacute progression and obvious absence of highrisk drug ingestion.^6^ Since there was a history of intake of itraconazole tablets for a week approximately 25 days before the disease manifestation, this case was managed in the line of TEN. There are few case reports of Itraconazole induced Steven Johnsons Syndrome (SJS)/TEN.^7^ However, in a study done in China for the epidemiology of SJS and TEN, Itraconazole does not come under very high risk/ offending drugs.^8^ Also, TEN encompasses flu-like prodrome, which was absent in this case, suggesting the possibility of other diagnoses too.^9^

It was quite a conundrum to diagnose the case as ACLE at presentation because there was no preceding diagnosis and family history of SLE, or any symptoms suggestive of SLE in the past. Diagnosis of SLE was made only after three days of hospital admission, based on SLICC criteria. Additionally, the TEN-like variant of SLE is a rare phenomenon, with very few cases reported worldwide. Therefore, it is likely that a physician misses diagnosing the case and treats in the line of TEN. In SLICC criteria, a toxic epidermal variant of SLE is included under Acute Cutaneous Lupus.

Hence, it is obvious that some peculiar drugs effective for ACLE could be missed when treated with an otherwise diagnosis. For example, Hydroxychloroquine (antimalarial) has been shown to have steroid-sparing effects and is administered as first-line therapy to most patients with systemic disease in SLE. The effects of Hydroxychloroquine on skin lesions like ACLE are especially beneficial. A 2017 meta-analysis found that antimalarials were 2.5 times more effective in lesions of ACLE compared with other lupus cutaneous skin lesion types.^10^ Adding this drug to the treatment regimen could give a better prognosis if the diagnosis is ACLE. If not, the same drug could aggravate TEN. There are cases of fatal TEN reported due to Hydroxychloroquine.^11^

The term "acute syndrome of apoptotic panepidermolysis" (ASAP) was proposed to include all the clinical situations of massive and acute epidermal cleavage resulting from apoptotic injury, irrespective of cause for diagnostic convenience. ASAP, then, comprises drug-induced TEN, TEN-like LE, and other conditions like acute graft versus host disease and TEN-like pseudoporphyria.^6^

Fortunately, the management plans for TEN and ACLE overlap greatly, albeit, with some peculiarity, and patients could benefit irrespective of diagnostic dilemma. Yet, holistic treatment guidelines for ASAP seem imperative for better prognosis and convenience of physicians irrespective of diagnostic dilemma.
